# Healthcare Policy Changes in Osteoporosis Can Improve Outcomes and Reduce Costs in the United States

**DOI:** 10.1002/jbm4.10192

**Published:** 2019-05-13

**Authors:** E Michael Lewiecki, Jesse D Ortendahl, Jacqueline Vanderpuye‐Orgle, Andreas Grauer, Jorge Arellano, Jeffrey Lemay, Amanda L Harmon, Michael S Broder, Andrea J Singer

**Affiliations:** ^1^ New Mexico Clinical Research & Osteoporosis Center Albuquerque NM USA; ^2^ Partnership for Health Analytic Research, LLC Beverly Hills CA USA; ^3^ Amgen Inc Thousand Oaks CA USA; ^4^ MedStar Georgetown University Hospital Washington DC USA

**Keywords:** OSTEOPOROSIS, FRACTURE PREVENTION, FRACTURE RISK ASSESSMENT, GENERAL POPULATION STUDIES, HEALTH ECONOMICS

## Abstract

In the United States, osteoporosis affects over 10 million adults, has high societal costs ($22 billion in 2008), and is currently being underdiagnosed and undertreated. Given an aging population, this burden is expected to rise. We projected the fracture burden in US women by modeling the expected demographic shift as well as potential policy changes. With the anticipated population aging and growth, annual fractures are projected to increase from 1.9 million to 3.2 million (68%), from 2018 to 2040, with related costs rising from $57 billion to over $95 billion. Policy‐driven expansion of case finding and treatment of at‐risk women could lower this burden, preventing 6.1 million fractures over the next 22 years while reducing payer costs by $29 billion and societal costs by $55 billion. Increasing use of osteoporosis‐related interventions can reduce fractures and result in substantial cost‐savings, a rare and fortunate combination given the current landscape in healthcare policy. © 2019 The Authors. JBMR Plus published by Wiley Periodicals, Inc. on behalf of American Society for Bone and Mineral Research.

## Introduction

From 1995 to 2005, the number of hip fractures in the United States declined substantially, which is thought to be due in part to increased awareness of the risk of falls, nutritional changes, screening of high‐risk patients to identify those who could benefit from treatment, and advances in treatments.(1) Recent data, however, suggest this trend may be reversing.(2) Although fractures in the elderly population may not receive the attention of other diseases such as cancer and cardiovascular disease, they can be devastating and carry a significant economic and clinical burden. In 2008, the direct medical costs related to osteoporotic fractures in the Medicare population was estimated to be $22 billion; however, this did not include indirect costs or fully account for the costs of long‐term care.(3) Many elderly individuals suffering fractures (especially of the hip) face lengthy hospitalizations, require long‐term care, lose independence, and have an increased risk of mortality in the following year. This economic and clinical burden is expected to be exacerbated by the aging of the US population, the recent trends of decreased bone density screening, a decrease in the treatment of high‐risk patients,(4,5) and uncertainties about future initiatives to promote bone health.

Although the future costs and rates of fractures are unknown, some of the anticipated burden could be avoided with appropriate identification and treatment of high‐risk women. Prevention of osteoporotic fractures is possible; diagnostic tools and effective treatments are available to help reduce the burden of disease, but they are underutilized. Although dual‐energy X‐ray absorptiometry (DXA) is highly effective at identifying at‐risk individuals(6) and recommended for all women age 65 years and older,(7) its utilization is low (11.3% in 2014).(5,8) This may be due in part to the decline in Medicare reimbursement of office‐based scans of about 70% since 2006, to levels below the cost of providing the procedure, resulting in the closure of some DXA facilities and limiting patient access to diagnostic services.(4,5) Additionally, despite the availability of pharmacologic therapies for preventing fractures,(9) treatment rates are low, even for the highest risk individuals. A Medicare analysis of 145,185 individuals with a fragility fracture found that only 30% received treatment over 12 months following the fracture, whereas a survey of women with a prior fracture estimated that the rate could be as low as 16%.(10–13) In order for the previous gains seen in fracture prevention to continue, preventive measures and therapies must be utilized effectively.

Expanding the use of healthcare services for osteoporosis may be difficult, given the under‐recognition of the risks, attribution of fractures to the normal course of aging, and a concern for treatment side effects. Additionally, the current environment prioritizes limiting short‐term expenses over long‐term cost savings. As longevity and the number of adults eligible for Medicare increases, there is a natural tendency to control costs; however, failing to increase preventive services and identify at‐risk individuals could result in a rise in fractures that adversely affects quality of life and increases costs.

To properly evaluate potential healthcare policies to reduce osteoporotic fractures, projections of the future rates and costs of these fractures are necessary. A range of trends could influence these projections, including the aging population, an increase in other risk factors for fracture, and changes in reimbursement for DXA. We therefore used a microsimulation forecasting model to incorporate these factors and assess a series of scenarios. Although previous studies have taken similar approaches, these studies are older, focused outside the US, did not account for certain cost components, or did not assess the impact of potential interventions.(14–18) This study fills the evidence gap. We aimed to project the future burden of osteoporotic fractures in women age 65 years and older (ie, those likely to be at high risk of fractures) and evaluate the impact of increasing efforts to prevent such fractures to inform future healthcare policy for this vulnerable population to improve outcomes.

## Materials and Methods

### Overview

We developed a microsimulation forecasting model to project the annual costs and incidence of osteoporotic fractures among US women age 65 years and older from 2018 to 2040. Fracture risk was estimated using the simplified form of the Fracture Risk Assessment Tool (FRAX),(19) a frequently used set of equations for estimating an individual's risk of fracture. This was populated with data on baseline characteristics and risk factors from the National Health and Nutrition Examination Survey (NHANES) which is a nationally‐representative population‐based survey conducted every 2 years.(20) Based on estimated risk and the impact of treatment (if applicable), individuals could experience fractures that would increase direct and indirect costs. Annual costs and fractures were compared across different scenarios of case finding and treatment rates attributable to potential policy changes.

### Fracture risk

To project whether each modeled individual experienced a fracture, we used the simplified chart version of FRAX developed at the University of Sheffield. FRAX, widely utilized in clinical practice, models a series of known risk factors for fracture, as well as age and bone mineral density (BMD) at the femoral neck, to estimate a 10‐year risk of hip fracture and a 10‐year risk of major osteoporotic fracture. The algorithms linking risk factors to fracture risk for the US differ by sex and vary among white, Hispanic, Black, and Asian populations. Although there are fracture risks that are not incorporated into FRAX (eg, obesity, diabetes), the tool is widely used in practice and has been previously validated. As the equation used to inform the full version of FRAX is not publicly available, and the tool on the website cannot be incorporated into our model, we relied on the FRAX simplified charts. The simplified charts use an individual's BMD and total number of clinical risk factors to predict fracture risk without accounting for the relative strength of each risk factor as a predictor. To assess the correlation between the simplified tables and the full version, we conducted an independent validation exercise using the NHANES sample, from which the risk as projected using the full FRAX algorithm and individual risk factor data were available.

Not all potential fracture sites are included in FRAX; it projects the risk of hip fracture individually, as well as major osteoporotic fracture, defined as clinical spine, forearm, hip, and shoulder. Although these sites comprise the sites most commonly associated with high costs, they do not include fractures in sites such as the pelvis, ribs, or foot. Therefore, we extrapolated the total number of fractures to include all potential sites. To do so, we adjusted the number of FRAX‐predicted fractures in our model by the estimated proportion of fractures included in FRAX among total fractures as observed in practice.(21)

### Risk factors

To estimate fracture risk for each individual assessed in the model, we generated population‐level estimates of risk factors using NHANES data. NHANES, conducted by the National Center for Health Statistics, assesses the health and nutritional status of adults and children, and tracks temporal changes. Using data from 2013‐2014, the most recent series that had all required information, we estimated the prevalence of the following widely accepted fracture risk factors: smoking; rheumatoid arthritis; long‐term glucocorticoid use; excessive alcohol use; parental history of a hip fracture; and previous fracture. Additionally, NHANES data were used to estimate mean BMD and the standard deviation.

### Other inputs

Estimates from NHANES were supplemented with data from published literature, publicly available databases, and subscription‐based pricing guides. The current and future number of women age 65 years and older, as well as the distribution by race, were based on estimates from the US Census Bureau.(22,23) Rates of DXA screening were based on a claims analysis that found 11.3% of women age 65 years and older received DXA in 2014.(5) Current treatment rates and trends were based on unpublished market share data, which indicated 9% of those ages 65 + years were treated (ie, filled at least one osteoporosis medication) in 2017, with the rate steadily declining since 2011. We made a simplifying assumption, based on unpublished market research, that treated patients would receive a “market basket” of currently available branded and generic agents. Treatment effectiveness of included drugs was based on a meta‐analysis of clinical trials and adjusted for adherence during each 1‐year period.(9,24,25) In the cited source(9) for efficacy and in the model, distinct estimates of treatment benefit were assumed for hip fractures and all other fractures. In scenarios that incorporated increased case finding, we assumed that 44% of all those assessed would subsequently receive treatment, consistent with prior estimates.(26)

Drug costs were based on wholesale acquisition costs from PriceRx,(27) whereas drug administration and DXA costs were based on a suggested range of fees for outpatient services.(28) Direct costs following a fracture were based on a claims analysis,(21) differed by category (ie, inpatient, outpatient, emergency department, long‐term care, pharmacy costs following a fracture, and other), and differed for individuals experiencing a single fracture within a year versus those with a subsequent fracture. Indirect costs attributable to productivity losses for the individual experiencing the fracture, and informal caregiving, were based on published estimates.(29,30) Costs from prior years were updated to 2018 $US using the Consumer Price Index (CPI) inflation calculator,(31) but to make results tangible to policymakers we did not apply discounting to future costs or clinical events.

### Microsimulation model

**Figure 1:**
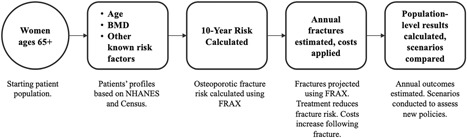
Model schematic

The model developed for this analysis was a microsimulation model programmed in Microsoft Excel 2018 (Microsoft Corp., Redmond, WA, USA). We assessed US women age 65 years and older, and estimated costs from payer and societal perspectives. For each calendar year from 2018 to 2040, hypothetical cohorts of 10 million women were simulated within each model scenario. Individuals entered the model with or without a history of fracture and were assigned an age, race, BMD, and risk factor profile. Based on these factors, 10‐year fracture risk was estimated and converted to an annual risk of fracture by dividing by 10, an approach that has been validated in a previous publication.(32) Random numbers between 0 and 1 were generated and compared to the annual risk to determine whether the individual experienced a fracture. If the number generated was less than the annual risk the individual was determined to have experienced a fracture, whereas if it was greater than the annual risk they did not. When determining whether an individual was designated for case finding and treatment, we assumed that those of highest risk as defined by FRAX would be prioritized. Although in practice it is likely the case that some individuals treated are at lower risk than other individuals who are untreated, our assumption of treating those at highest risk should be the goal, and is likely closer to reality than assuming treatment is randomly distributed. Those receiving treatment had their fracture risk reduced by the magnitude of treatment benefit, as described in the model inputs section. A visual depiction of the flow of individuals through the model is shown in Fig. 1. Common random numbers were used to reduce the impact of stochastic variability, such that differences between calendar years were driven solely by demographic shifts.(33)

### Analyses

In the base case, we estimated the annual payer and societal costs, as well as the cumulative costs over 5‐year, 7‐year, 10‐year, 15‐year, and 22‐year periods. The costs without further interventions (ie, only accounting for population aging and growth), were compared with scenarios in which policies were enacted that encourage an increase in either case finding, drug therapy, or both. In scenarios with increased case finding, 44% of all women undergoing DXA received subsequent treatment based on a published estimate.(26) We used DXA scanning as a proxy for any method of identifying patients at high‐risk for fracture who could be eligible for treatment. In scenarios with increased treatment, we assumed that the proportion of those treated among those identified as being candidates for treatment increased. We also projected the total number of fractures under each scenario to estimate the clinical and economic implications of increased preventive services. In addition to comparing these scenarios, we conducted a series of one‐way and multiway sensitivity analyses to assess the impact of structural and parameter uncertainty on model results.

## Results

The analysis of NHANES data showed that among elderly women, the proportion of individuals who have experienced a previous fracture decreased from 2003‐2014, as did the average BMD. The proportion consuming more than three drinks per day increased over the same period, as did the proportion of individuals with a history of glucocorticoid use. Other clinical risk factors remained relatively constant throughout the observation period. In the validation exercise we conducted, 10‐year FRAX scores reported using the full form of the equation were within 1% of those derived from the simplified tables, indicating reasonable correspondence.

Using the validated FRAX tables and insights from NHANES to estimate the future burden among Medicare‐eligible women, we found that total fractures and fracture‐related costs will increase substantially under the status quo of underdiagnosis and undertreatment of osteoporosis (Exhibits 2‐4).(5,8) Due to an aging and growing population, annual number of fractures is expected to increase 68%, from 1.9 million to over 3.2 million, similar to findings from a claims‐based analysis.(34) Over the next 22 years, this amounts to 61.6 million fractures, of which approximately 10% will be hip fractures.

Correspondingly, the annual direct medical costs associated with fractures were estimated to be $48.8 billion in 2018 and to increase to $81.5 billion in 2040. When relevant indirect societal costs related to productivity losses and informal caregiving were included, annual costs increased to $57.0 billion in 2018 and $95.2 billion in 2040.

**Figure 2:**
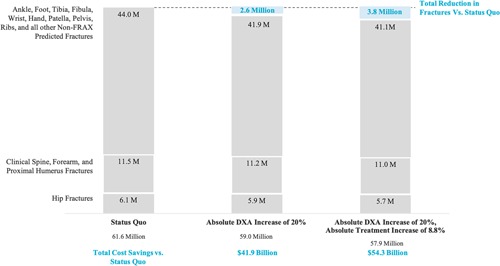
Fractures by site from 2018 to 2040 with increased case finding and treatment. notes. DXA = dual‐energy X‐ray absorptiometry; M = millions

If interventions that increase case finding were implemented, the economic and clinical burden could be mitigated. We found that an absolute increase in case finding of 20% would prevent 2.6 million fractures over the next 22 years, and a 50% absolute increase would prevent over 4 million fractures over the same time period. An increase in case finding of 20%, leading to 31.3% of women undergoing DXA and a subset of those receiving treatment, would reduce total cumulative costs from 2018 to 2040 by $41.9 billion. If case finding increased by 50%, such that 61.3% of the population was scanned and 31% were treated, costs would decrease by $45.9 billion. Of these savings, approximately 60% are direct medical costs, while 40% are indirect costs. Notably, even with increases in costs of diagnosis and treatment associated with increased case finding, preventive services would still represent less than 6% of total costs of osteoporosis (Fig. 2).

Assuming treatment initiation and adherence were increased among newly identified individuals, the clinical and economic benefits would further increase. If case finding were to increase to 31.3% of the population (20% increase) and twice as many of the individuals identified as being eligible for treatment were treated, we projected that 3.7 million fractures could be prevented leading to $54.3 billion decreased costs to society over the next 22 years. In a scenario with an absolute DXA increase of 50% and twice as many patients being treated, 6.1 million fractures would be prevented from 2018 to 2040, with $28.6 billion of savings in Medicare spending and a total reduction in costs of $54.5 billion when including indirect costs (Table 1).

**Table 1:** Clinical and Economic Outcomes Under the Status Quo and With Increased Utilization of Case Finding and Treatment

Outcomes	2018–2023	2018–2025	2018–2028	2018–2033	2018–2040
Fractures					
Status quo (11.3% case finding, 9% treatment)	10,339,728	17,437,985	22,558,073	36,598,591	61,603,120
31.3% case finding, 17.8% treatment^a^					
Total	9,973,404	16,792,424	21,701,435	35,124,422	59,007,139
Difference versus status quo	366,324	645,561	856,638	1,474,170	2,595,981
31.3% case finding, 26.6% treatment^b^					
Total	9,781,843	16,472,304	21,291,346	34,461,482	57,904,438
Difference vs. Status Quo	557,885	965,681	1,266,727	2,137,109	3,698,682
Costs ($billions)					
Status quo (11.3% case finding, 9% treatment)	$305.2	$514.4	$665.2	$1,078.5	$1,813.7
31.3% case finding, 17.8% treatment^a^					
Total	$299.8	$504.7	$652.1	$1,055.2	$1,771.8
Difference versus status quo	$5.4	$9.7	$13.1	$23.3	$41.9
31.3% case finding, 26.6% treatment^b^					
Total	$297.6	$501.1	$647.6	$1,047.8	$1,759.4
Difference versus status quo	$7.6	$13.3	$17.6	$30.7	$54.3

^a^ It is assumed that among all women newly scanned with increased case finding, 44% subsequently received treatment.

^b^ In scenarios with increased case finding and treatment, the proportion of those receiving treatment following a scan was increased from 44% to 88%.

**Figure 3:**
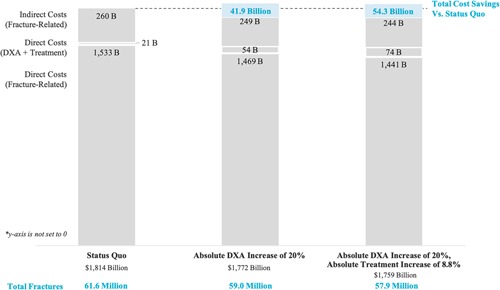
Direct and indirect costs from 2018 to 2040 with increased case finding and treatment. DXA = dual‐energy X‐ray absorptiometry; B = billions

In one‐way sensitivity analyses we found generally that results were robust and directionally consistent; ie, the findings of reduced fractures and cost savings were maintained. Results were most sensitive to shifts in the direct medical costs of caring for patients after a fracture and assumptions related to treatment effectiveness (ie, adherence, compliance, efficacy). When assuming that observed trends in fracture risk factors continued, the total fractures and costs were approximately 4% to 5% lower across all scenarios. Although DXA is currently performed more frequently in the office setting than in the hospital,(35) as modeled in our base case assessment, we also considered the impact of altering the DXA reimbursement rate in the office setting to match the reimbursement in the hospital setting. This increased reimbursement could lead to wider availability of DXA, and is an example of a potential policy change that could lead to the increased utilization we are modeling. In the scenario in which DXA was reimbursed at the rate it is in the hospital setting, DXA utilization was increased to 31.3% of individuals, and treatment doubled to 26.6% of women ages 65 years and older, we found that there was still clinical benefit and cost savings with increased case finding and treatment, but the cumulative savings from 2018 to 2040 were reduced from $54.3 billion to $40.9 billion. (Fig. 3)

## DISCUSSION

As a result of longer lifespans, growth in the Medicare population, and underdiagnosis and undertreatment of high‐risk women, we project both fractures and fracture‐related costs to increase by nearly 70% by 2040 in the United States. However, this dire outcome is avoidable with a variety of changes that will simultaneously reduce fractures and costs while improving quality of life for millions of older Americans, a unique opportunity in the current landscape. The money saved by these changes could then be reallocated to treating other diseases, providing an immense ancillary benefit in a healthcare system where rising costs are a major challenge.

Many potential policy interventions that increase diagnosis and treatment for osteoporosis have been identified and shown to be effective on a smaller scale in the United States. One such approach is the implementation of quality measures linking higher reimbursement for increased use of beneficial interventions. There has been discussion of creation and adoption of quality metrics in osteoporosis to encourage appropriate use of DXA and treatment in patients at high risk, such as those who have experienced a prior fracture, those with other clinical risk factors, and those with low BMD. General practitioner‐focused interventions that highlighted the importance of evaluating fracture risk increased DXA use and osteoporosis treatment by nearly 50%.(36–38) We used this estimate as the basis for our scenario in which there was assumed to be an absolute DXA increase of 50%.

Organizing clinicians to work in multidisciplinary teams, having a care coordinator, or implementing chronic disease management pathways that focus on osteoporosis and fractures could also help reduce fragility fractures. Adding a case manager or care coordinator to the care team can increase BMD testing, treatment initiation, and treatment adherence by up to 30%.(39,40) This served as the basis for our more conservative assessment of a potential 20% absolute increase in DXA. Additionally, Fracture Liaison Services (FLS) have been incorporated into some health systems and are effective; a model‐based analysis found FLS to reduce costs and improve clinical outcomes.(41) Even without shifting or adding providers, simply encouraging participation in osteoporosis‐related continuing medical education could increase use of preventative services.(42)

In rural or resource‐poor settings where DXA may be unavailable, there would be benefit to identifying high‐risk individuals without requiring BMD testing (eg, those with prior fragility fractures), and encouraging use of the version of FRAX that uses body mass index as opposed to BMD.(43) Additionally, although FRAX is widely used and validated, development of additional quantitative tools to help predict immediate and long‐term fracture risk could encourage more efficient use of treatment.(44) Finally, there might be potential benefit to the increased use of quantitative ultrasound devices as screening tools to assess fracture risk.(45)

Interventions that both improve outcomes and quality of life and simultaneously reduce costs are uncommon. In cost‐effectiveness analyses, one would describe increased case finding and treatment as a dominant strategy compared with the status quo (eg, additional prevention is both clinically and economically superior). A systematic review of the cost‐effectiveness literature found less than 20% of reviewed articles assessing preventive services had a dominant strategy,(46) whereas another study found that in only 2% of oncology publications did one option dominate another.(47) Given the debates over how much society should be willing to pay to increase health, any opportunity to save money while improving health should be seized.

This study was unique in that it projected both clinical and economic outcomes related to osteoporotic fractures in the United States, and it assessed the impact of interventions to reduce the burden. Previously published osteoporosis projection models have all differed in their approaches. A prior US‐based study made economic and clinical projections(14); however, the projection period was limited to 2005‐2025, and the projections were based solely on population shifts without consideration of interventions to change diagnosis and treatment rates. A Canadian publication assessed the long‐term cost effectiveness of various pharmacological treatments(15) but did not consider policy levers or changes beyond drug innovations. A microsimulation model was used to project the lifetime risk of osteoporotic fractures in Belgium(16); however, it assessed a single cohort over their lifetime and did not incorporate costs. A study similar to ours included both direct and indirect costs and projected outcomes to 2050(17); however, it was based in Germany. Another study made projections of osteoporosis burden and costs to 2050(18) but was based in China, did not consider indirect costs, and made projections by multiplying current costs by changes in population as opposed to including further changes in risk factors or interventions. These prior studies have projected the burden, both within and outside the United States, using different approaches and have consistent findings of an expected increase in fractures related to shifting demographics.

Although this analysis followed modeling guidelines and relied on the best available data, there are some limitations. In any model‐based analysis, especially when making projections as far out as 2040, uncertainty exists and simplifying assumptions must be made. However, we erred on the conservative side, underestimating the results to provide a lower bound on the potential benefits of increased case finding and treatment. We assumed costs would remain constant in the future, which has not been the case historically. To the extent that healthcare costs continue rising, the growth in projected spending would be an underestimate. We did not attempt to quantify the clinical benefits in terms of quality‐adjusted life years, and this could be an area of future research. We also did not assess the impact of increased treatment in men, as fractures are more common in postmenopausal women. Inclusion of men in this analysis would have increased the potential fractures prevented with increased case finding and treatment. Our assumption that those at highest risk would be identified and treated first might not reflect reality, although it should be the goal in clinical practice. Additionally, we did not consider socioeconomic differences or other disparities that might impact utilization by subgroups. In the model, an increase in case finding was incorporated by increasing the rate of DXA, although other methods have also been shown to be effective. To the extent case finding can occur without the need for DXA, the cost savings found in scenarios with increased case finding could be an underestimate. In estimating the treatment effectiveness, we assumed that all fractures could be prevented at rates shown in meta‐analyses; however, treatment might not prevent fractures to all potential sites at the same rate as measured in clinical trials. Additionally, we only considered currently available treatments. To the extent that new treatment alternatives are introduced that are more efficacious, the fracture reduction and cost savings in scenarios with increased utilization would be an underestimate of the true benefit. We did not consider any impact of overdiagnosis or adverse events associated with treatment; however, these could be considered in future studies. The risk of fractures was estimated using the simplified charts from FRAX, and although they have been well validated, they are not perfect predictors.

Though projections of fracture rates and costs in the absence of further action are alarming, we found that increasing identification of high‐risk individuals and providing effective treatments could reduce the clinical burden while saving costs and provide valuable policy solutions to address this at‐risk population. An emphasis on bone health by policymakers, payers, and clinicians could improve health outcomes for elderly women while efficiently utilizing healthcare services.

## Disclosures

EML has received institutional grant/research support from Radius, Amgen, PFEnex, and Mereo; he has served on scientific advisory boards or consulted for Amgen, Radius, Alexion, Ultragenyx, Sandoz, and Celltrion; he serves on the speakers’ bureau for Radius and Alexion; he is a board member of the National Osteoporosis Foundation, International Society for Clinical Densitometry, and Osteoporosis Foundation of New Mexico. JDO, ALH, and MSB are employees of the Partnership for Health Analytic Research, LLC, which received payment for conducting the analyses described in this manuscript. AJS has served on scientific advisory boards or consulted for Agnovos, Amgen, Eli Lilly, Merit, Radius, and UCB; she is on the speakers’ bureau for Amgen, Eli Lilly and Radius; she is a trustee of the National Osteoporosis Foundation (non‐remunerative position). JA and JL are full‐time employees of Amgen and own stock and stock options. AG and JVO were full‐time employees of Amgen when this study was conducted and own stock and stock options.

## References

[1] Brauer CA , Coca‐Perraillon M , Cutler DM , Rosen AB. Incidence and mortality of hip fractures in the United States. JAMA. 2009;302(14):1573–9. 19826027 10.1001/jama.2009.1462 PMC4410861

[2] Lewiecki EM , Wright NC , Curtis JR. et al. Hip fracture trends in the United States, 2002 to 2015. Osteoporos Int. 2018;29(3):717–22. 29282482 10.1007/s00198-017-4345-0

[3] Blume SW , Curtis JR. Medical costs of osteoporosis in the elderly Medicare population. Osteoporos Int. 2011;22(6):1835–44. 21165602 10.1007/s00198-010-1419-7 PMC3767374

[4] King AB , Fiorentino DM. Medicare payment cuts for osteoporosis testing reduced use despite tests’ benefit in reducing fractures. Health Aff (Millwood) 2011;30(12):2362–70. 22147865 10.1377/hlthaff.2011.0233

[5] Lewiecki EM , Adler R , Curtis J et al. Hip fractures and declining DXA testing: at a breaking point? J Bone Miner Res 2016;31(S1):S1–411.

[6] Office of the Surgeon General (US) . Bone Health and Osteoporosis: A Report of the Surgeon General [Internet]. Rockville (MD): Office of the Surgeon General (US); 2004 [cited 2019 Mar 24]. (Reports of the Surgeon General). Available from: http://www.ncbi.nlm.nih.gov/books/NBK45513/. 20945569

[7] U.S. Preventive Services Task Force Screening for osteoporosis: U.S. preventive services task force recommendation statement. Ann Intern Med 2011;154(5):356–64. 21242341 10.7326/0003-4819-154-5-201103010-00307

[8] Gillespie CW , Morin PE. Trends and disparities in osteoporosis screening among women in the United States, 2008‐2014. Am J Med 2017;130(3):306–16. 27884649 10.1016/j.amjmed.2016.10.018

[9] Freemantle N , Cooper C , Diez‐Perez A et al. Results of indirect and mixed treatment comparison of fracture efficacy for osteoporosis treatments: a meta‐analysis. Osteoporos Int. 2013;24(1):209–17. 22832638 10.1007/s00198-012-2068-9 PMC3662000

[10] Yusuf AA , Matlon TJ , Grauer A , Barron R , Chandler D , Peng Y. Utilization of osteoporosis medication after a fragility fracture among elderly Medicare beneficiaries. Arch Osteoporos 2016;11(1):31. Dec 1 27696099 10.1007/s11657-016-0285-0

[11] Iba K , Takada J , Hatakeyama N et al. Underutilization of antiosteoporotic drugs by orthopedic surgeons for prevention of a secondary osteoporotic fracture. J Orthop Sci 2006;11(5):446–9. 17013730 10.1007/s00776-006-1050-9

[12] Andrade SE , Majumdar SR , Chan KA et al. Low frequency of treatment of osteoporosis among postmenopausal women following a fracture. Arch Intern Med 2003;163(17):2052–7. 14504118 10.1001/archinte.163.17.2052

[13] Boudreau DM , Yu O , Balasubramanian A et al. A survey of women's awareness of and reasons for lack of postfracture osteoporotic care. J Am Geriatr Soc 2017;65(8):1829–35. 28422273 10.1111/jgs.14921

[14] Burge R , Dawson‐Hughes B , Solomon DH , Wong JB , King A , Tosteson A. Incidence and economic burden of osteoporosis‐related fractures in the United States, 2005‐2025. J Bone Miner Res 2006;22(3):465–475. 10.1359/jbmr.061113 17144789

[15] Goeree R , Blackhouse G , Adachi J. Cost‐effectiveness of alternative treatments for women with osteoporosis in Canada. Curr Med Res Opin 2006;22(7):1425–36. 16834841 10.1185/030079906X115568

[16] Hiligsmann M , Bruyère O , Ethgen O , Gathon H‐J , Reginster J‐Y. Lifetime absolute risk of hip and other osteoporotic fracture in Belgian women. Bone. 2008;43(6):991–4. 18817901 10.1016/j.bone.2008.08.119

[17] Konnopka A , Jerusel N , König H‐H. The health and economic consequences of osteopenia‐ and osteoporosis‐attributable hip fractures in Germany: estimation for 2002 and projection until 2050. Osteoporos Int. 2009;20(7):1117–29. 19048180 10.1007/s00198-008-0781-1

[18] Si L , Winzenberg TM , Jiang Q , Chen M , Palmer AJ. Projection of osteoporosis‐related fractures and costs in China: 2010–2050. Osteoporos Int. 2015;26(7):1929–37. 25761729 10.1007/s00198-015-3093-2

[19] Centre for Metabolic Bone Diseases , University of Sheffield, UK. FRAX® Fracture Risk Assessment Tool [Internet]. [cited 2019 Mar 24]. Available from: https://www.sheffield.ac.uk/FRAX/.

[20] Centers for Disease Control and Prevention . NHANES ‐ National Health and Nutrition Examination Survey [Internet]. [cited 2019 Mar 24]. Available from: https://www.cdc.gov/nchs/nhanes/.

[21] Weaver J , Sajjan S , Lewiecki EM , Harris ST , Marvos P. Prevalence and cost of subsequent fractures among U.S. patients with an incident fracture. J Manag Care Spec Pharm 2017;23(4):461–71. 28345441 10.18553/jmcp.2017.23.4.461 PMC10398116

[22] Colby SL , Ortman JM. Projections of the size and composition of the U.S. population: 2014 to 2060. Washington, DC: US Census Bureau; 2014. Current Population Reports, P25‐1143:13.

[23] U.S. Census Bureau [Internet] . Available from: https://www.census.gov/.

[24] Imaz I , Zegarra P , González‐Enríquez J , Rubio B , Alcazar R , Amate JM. Poor bisphosphonate adherence for treatment of osteoporosis increases fracture risk: systematic review and meta‐analysis. Osteoporos Int. 2010;21(11):1943–51. 19967338 10.1007/s00198-009-1134-4

[25] Durden E , Pinto L , Lopez‐Gonzalez L , Juneau P , Barron R. Two‐year persistence and compliance with osteoporosis therapies among postmenopausal women in a commercially insured population in the United States. Arch Osteoporos 2017;12(1):22. 28243883 10.1007/s11657-017-0316-5 PMC5329075

[26] King AB , Saag KG , Burge RT , Pisu M , Goel N. Fracture Reduction Affects Medicare Economics (FRAME): Impact of increased osteoporosis diagnosis and treatment. Osteoporos Int. 2005;16(12):1545–57. 15942702 10.1007/s00198-005-1869-5

[27] Wolters Kluwer. Price Rx [Internet] . 2018 [cited 2019 Mar 24]. Available from: https://pricerx.medispan.com/.

[28] 2018 Physicians’ Fee & Coding Guide . InHealth; 2018.

[29] Pike C , Birnbaum HG , Schiller M , Sharma H , Burge R , Edgell ET. Direct and indirect costs of non‐vertebral fracture patients with osteoporosis in the US. PharmacoEconomics. 2010;28(5):395–409. 20402541 10.2165/11531040-000000000-00000

[30] Vanness DJ , Tosteson ANA. Estimating the opportunity costs of osteoporosis in the United States. Top Geriatr Rehabil 2005;21(1):4–16.

[31] United States Department of Labor , Bureau of Labor Statistics. CPI (Consumer Price Index) Inflation Calculator [Internet]. 2018 [cited 2019 Mar 24]. Available from: http://www.bls.gov/data/inflation_calculator.htm.

[32] Leslie WD , Majumdar SR , Morin SN et al. FRAX for fracture prediction shorter and longer than 10 years: the Manitoba BMD registry. Osteoporos Int. 2017;28(9):2557–64. 28593449 10.1007/s00198-017-4091-3

[33] Stout NK , Goldie SJ. Keeping the noise down: common random numbers for disease simulation modeling. Health Care Manag Sci 2008;11(4):399–406. 18998599 10.1007/s10729-008-9067-6 PMC2761656

[34] Leader D, Jr , Williams SA , Curtis JR , Gut R. Osteoporosis‐related fracture events in the U.S. AMCP Nexus; 2017 Oct 16; Dallas, TX, USA.

[35] Zhang J , Delzell E , Zhao H et al. Central DXA utilization shifts from office‐based to hospital‐based settings among Medicare beneficiaries in the wake of reimbursement changes. J Bone Miner Res 2012;27(4):858–64. 22190195 10.1002/jbmr.1534

[36] Laliberté M‐C. , Perreault S , Dragomir A et al. Impact of a primary care physician workshop on osteoporosis medical practices. Osteoporos Int. 2010;21(9):1471–85. 19937428 10.1007/s00198-009-1116-6

[37] Majumdar SR , Johnson JA , McAlister FA et al. Multifaceted intervention to improve diagnosis and treatment of osteoporosis in patients with recent wrist fracture: a randomized controlled trial. CMAJ. 2008;178(5):569–75. 18299546 10.1503/cmaj.070981 PMC2244663

[38] Cranney A , Wells GA , Yetisir E et al. Ibandronate for the prevention of nonvertebral fractures: a pooled analysis of individual patient data. Osteoporos Int. 2009;20(2):291–7. 18663402 10.1007/s00198-008-0653-8

[39] Miki RA , Oetgen ME , Kirk J , Insogna K L , Lindskog DM. Orthopaedic management improves the rate of early osteoporosis treatment after hip fracture. A randomized clinical trial. J Bone Joint Surg Am 2008;90(11):2346–53. 18978403 10.2106/JBJS.G.01246

[40] Majumdar SR , Beaupre LA , Harley CH et al. Use of a case manager to improve osteoporosis treatment after hip fracture: results of a randomized controlled trial. Arch Intern Med 2007;167(19):2110–5. 17954806 10.1001/archinte.167.19.2110

[41] Solomon DH , Patrick AR , Schousboe J , Losina E. The potential economic benefits of improved postfracture care: a cost‐effectiveness analysis of a fracture liaison service in the US health‐care system. J Bone Miner Res 2014;29(7):1667–74. 24443384 10.1002/jbmr.2180 PMC4176766

[42] Greenspan SL , Bilezikian JP , Watts NB et al. A Clinician performance initiative to improve quality of care for patients with osteoporosis. J Womens Health 2013;22(10):853–61. 10.1089/jwh.2013.4388 PMC3837565 24011023

[43] Goldshtein I , Gerber Y , Ish‐Shalom S , Leshno M Fracture risk assessment with FRAX using real‐world data in a population‐based cohort from Israel. Am J Epidemiol 2018;187(1):94–102. 28520844 10.1093/aje/kwx128

[44] Bonaccorsi G , Messina C , Cervellati C et al. Fracture risk assessment in postmenopausal women with diabetes: comparison between DeFRA and FRAX tools. Gynecol Endocrinol. 2018;34(5):404–8. 29172781 10.1080/09513590.2017.1407308

[45] Schousboe JT , Riekkinen O , Karjalainen J Prediction of hip osteoporosis by DXA using a novel pulse‐echo ultrasound device. Osteoporos Int. 2017;28(1):85–93. 27492435 10.1007/s00198-016-3722-4

[46] Cohen JT , Neumann PJ , Weinstein MC. Does preventive care save money? Health economics and the presidential candidates. N Engl J Med 2008;358(7):661–3. 18272889 10.1056/NEJMp0708558

[47] Winn AN , Ekwueme DU , Guy GP , Neumann PJ Cost‐utility analysis of cancer prevention, treatment, and control. Am J Prev Med 2016;50(2):241–8. 26470806 10.1016/j.amepre.2015.08.009 PMC5846573

